# Adapting organizational culture scale into healthcare professional education: a scale validity and reliability analysis

**DOI:** 10.1186/s12960-025-01006-2

**Published:** 2025-07-15

**Authors:** Aysel Başer, Ömer Faruk Sönmez, Duygu Kürklü Arpaçay, Hatice Şahin

**Affiliations:** 1 Department of Medical Education, Faculty of Medicine, Izmir Democracy University, İzmir, Türkiye; 2 School of Medicine and Population Health, University of Sheffield, Sheffield, UK; 3 Department of Prosthodontics, Faculty of Dentistry, Izmir Tınaztepe University, İzmir, Türkiye; 4 Department of Medical Education, Faculty of Medicine, Ege University, İzmir, Türkiye

**Keywords:** Organizational culture scale, Healthcare professional education, Scale adaptation, Exploratory factor analysis, Confirmatory factor analysis, Reliability analysis

## Abstract

**Background:**

Organizational culture significantly influences the quality of healthcare services and healthcare professional education. Although various scales exist to measure organizational culture at the undergraduate level, validated instruments specifically tailored for healthcare professional education remain scarce. The study aims to validate the adapted scale and provide empirical insights into organizational culture in healthcare professional education.

**Methods:**

The adaptation process involved expert consultations to ensure content and face validity, followed by a mixed-methods approach. Quantitative data were collected from 402 students enrolled in the Faculties of Medicine, Dentistry, and Health Sciences during the 2023–2024 academic year. Exploratory and confirmatory factor analyses were performed to examine the scale’s structure. Reliability was assessed using Cronbach’s alpha and McDonald’s omega coefficients.

**Results:**

Expert review led to the removal of redundant and unclear items, refining the scale to 30 items across five subscales: Structural Order and Formality, Belonging and Collective Responsibility, Achievement and Performance Orientation, Authority and Hierarchy, and Competition Orientation. The Kaiser–Meyer–Olkin measure (0.846) and Bartlett’s test confirmed sample adequacy. Exploratory factor analysis explained 40% of the total variance. Confirmatory factor analysis showed acceptable model fit indices (*χ*^*2*^/df = 3.37, RMSEA = 0.091, CFI = 0.95, TLI = 0.94). The scale demonstrated strong internal consistency (overall McDonald’s *ω* = 0.878; Cronbach’s *α* = 0.874), although lower reliability was noted for Authority and Hierarchy and Competition Orientation subscales.

**Conclusions:**

The adapted organizational culture scale is a valid and reliable tool for assessing organizational culture in healthcare professional education. Its use is recommended for tracking cultural changes and supporting strategic educational improvements. Further validation across different institutions and cultural contexts is encouraged to reinforce its generalizability.

**Supplementary Information:**

The online version contains supplementary material available at 10.1186/s12960-025-01006-2.

## Background

The term organizational culture finds its roots in anthropology from the post-Taylorism era of the 1930s, initially as a facet of human relations. Its application within the healthcare sector was minimal until Pettigrew’s pivotal 1979 work, On Studying Organizational Cultures [1, 2]. The literature often uses “organizational culture” and “organizational climate” interchangeably, yet they hold distinct meanings [3]. Organizational culture, which is crucial for guiding actions and interpretations within an organization, is built on fundamental assumptions and values, and plays a significant role in improving healthcare quality [4]. While organizational climate’s tangible dimensions allow easier measurement, the abstract nature of organizational culture’s values and beliefs presents assessment challenges. In undergraduate institutions that educate healthcare professionals, the need to assess organizational culture arises from its significant influence not only on work life and care quality, but also on the effectiveness of professional education [1, 5].

The education of healthcare professionals involves more than the transmission of knowledge and skills; it also encompasses organizational and cultural processes such as teamwork, ethical responsibility, role modeling, institutional belonging, and interprofessional collaboration. In this context, healthcare professional education is not limited to individual learning but takes place within institutional structures that sustain and shape collective values and cultural norms. The literature highlights that effective communication and collaboration among professionals from diverse cultural backgrounds significantly influence healthcare outcomes [6]. Therefore, identifying the prevailing organizational culture within healthcare professional education environments is essential for improving educational quality, enhancing learning environments, and supporting professional identity formation [7].

Organizational culture is a multidimensional structure that influences various domains, including knowledge management, psychological well-being, communication quality, and leadership impact. Literature suggests that organizational culture positively affects knowledge-sharing processes, reduces medical errors, and supports institutional learning environments [8, 9]. Moreover, it directly impacts the psychological well-being and occupational engagement of healthcare professionals [10]. Other studies indicate that organizational culture plays a crucial role in shaping nursing students’ willingness to report errors and their sense of professional responsibility [1]. These findings demonstrate that organizational culture is not only significant in clinical service delivery, but also in determining the structure, quality, and atmosphere of professional education.

On the other hand, perceptions of organizational culture may vary across different departments within an institution, potentially affecting institutional development capacity and the safety culture. The literature suggests that such differences can negatively affect institutional functioning and the learning environment, while also emphasizing that leadership plays a pivotal role in shaping organizational culture and that a positive culture significantly enhances employee engagement [11, 12]. It is evident that undergraduate-level institutions that educate healthcare professionals are influenced by organizational culture. This influence extends beyond administrative processes, as it also plays a pivotal role in reinforcing faculty role modeling, fostering student–institution affiliation, and enhancing the overall quality of educational interaction.

While widely used models—such as Hofstede’s socio-cultural value framework (e.g., power distance, uncertainty avoidance, individualism–collectivism) [3], Denison’s performance and adaptation-based model [13], and Cameron & Quinn’s competing values typology [14]—offer valuable insights into organizational dynamics, they fall short in capturing the specific cultural dimensions relevant to healthcare professional education. These models are limited in addressing critical aspects such as interprofessional collaboration, institutional belonging, role modeling, and mentor–apprentice relationships. In contrast, Pheysey’s four-dimensional model of organizational culture—comprising power, role, achievement, and support—provides a more comprehensive theoretical basis for understanding cultural orientations within undergraduate-level institutions that educate healthcare professionals [15].

There is an increasing necessity to emphasize organizational culture as a key factor in evaluating educational programs and gaining a more advanced understanding of the learning in which future healthcare professionals receive their education. The quality of educational experiences is not only influenced by organizational culture, but also by professional identity formation, interprofessional collaboration, and the development of ethical and leadership competencies. Despite the recognized relevance of organizational culture in educational contexts, empirical tools, specifically designed to capture the cultural dynamics of undergraduate-level institutions in healthcare remain limited. This study addresses this gap by investigating organizational culture in these institutions through a theoretically grounded and contextually adapted framework. It aims to validate the adapted instrument and contribute to the empirical understanding of how organizational culture manifests in settings that educate future healthcare professionals—an area where such insights are essential for fostering academic engagement, professional identity formation, and institutional effectiveness.

## Methods

This descriptive methodological study aims to assess the validity and reliability of a culturally adapted Organizational Culture Scale (OCS) for use in undergraduate institutions that train healthcare professionals.

### Instrument selection and theoretical justification

In this study, we employed the organizational culture measurement tool developed by İpek, which is grounded in Diana Pheysey’s typology of organizational culture [15–17]. Although widely recognized models—such as those proposed by Hofstede, Cameron and Quinn, and Denison and Mishra—have been extensively reviewed in the literature, these frameworks are predominantly oriented toward assessing institutional performance or sector-specific values and are typically applied within healthcare service organizations [3, 13, 14]. Among various models, Pheysey’s framework offers a theoretical basis aligned with the needs of healthcare education. However, İpek’s adaptation was preferred for this study due to its stronger cultural relevance and empirical validation within the Turkish context [15]. As such, they are not readily transferable to the structural and pedagogical dynamics of institutions dedicated to healthcare professional education. These models often fail to capture essential educational components such as interprofessional collaboration, institutional belonging, role modeling, and mentor–apprentice relationships—elements that are integral to the formation of professional identity in healthcare settings. Consequently, İpek’s scale was chosen over Pheysey’s original framework due to its stronger theoretical coherence, inclusion of culturally relevant dimensions tailored to the healthcare education context, and its established validity and reliability as a standardized instrument in Turkish.

Originally tailored for secondary education, the OCS comprises four distinct subscales—power culture, role culture, achievement culture, and support culture—across 36 items using a 5-point Likert scale and incorporates constructs that align closely with the goals and environment of healthcare professional education, where factors such as teamwork and societal role modeling are emphasized [17].

Thus, a theoretically grounded and contextually adapted measurement tool was obtained, enabling the assessment of the unique organizational dynamics of undergraduate-level institutions that provide healthcare professional education.

This study method employed the following steps in adapting the OCS to healthcare professional education:

### Expert consultation for scale adaptation

For the adaptation of the scale, experts were engaged from various health professional education disciplines among university lecturers and experienced researchers. To validate the content and establish face validity, consultations were sought from experts in medicine (*n* = 2), dentistry (*n* = 1), and nursing (*n* = 1), utilizing the Lawshe technique for consensus. During consultation phase, it was determined that two scale items (numbers 5 and 29) were redundant or unclear, leading to their removal. Consequently, the adapted scale (before analysis) comprised 34 items for further analysis.

### Data collection

Participants selection: adhering to established practice in scale adaptation research, the study employed a methodology for sample size calculation, referencing the widely recognized ‘tens’ rule by Tabachnick and Fidell [18, 19]. This principle guided the recruitment of a sample size at least tenfold the scale’s item count, targeting a minimum of 360 participants to ensure robust statistical power. Participation was strictly voluntary with informed consent.

Inclusive recruitment: This study included students from the Faculties of Medicine and Dentistry, as well as the Departments of Nutrition, Physiotherapy, Nursing, and Sports within the Faculty of Health Sciences, and the Vocational School of Health Services at a public university during the 2023–2024 academic year, ensuring a diverse and comprehensive participant pool.

Data collection instruments: Data were collected in two parts: the OCS and a sociodemographic (age, gender, grade, enrolled program) questionnaire capturing essential participant information.

### Factor analysis

During the factor analysis stage, comprehensive exploratory and confirmatory analyses were performed to ensure the scale’s construct validity, adhering to the guidelines established by Pett [20]. A five-step exploratory factor analysis process, as outlined by Williams, was employed [19]. The data’s suitability for factor analysis was confirmed through Kaiser–Meyer–Olkin (KMO) and Bartlett’s tests [19].

Principal component analysis was employed for initial factor extraction, with oblique rotation subsequently applied to refine the factor structure accurately [21]. The item selection process was guided by the Eigenvalue > 1 Rule, aiming for a cumulative variance explanation within the optimal range of 40–60% [19–22]. The Oblique Oblimin rotation method was then utilized to further clarify factor loadings [19]. Following the application of the Oblique Oblimin rotation, four items (Items 5, 29, 2, and 14) with factor loadings below 0.10 were initially removed for their minimal contribution to the scale’s structure. [23]. Specifically, two items (Items 5 and 29) were eliminated during the expert consultation phase for being redundant or unclear; two more (Items 2 and 14) items were excluded due to low factor loadings during EFA; and an additional two items (Items 31 and 33) were removed in the interpretation stage due to a lack of conceptual fit with any factor. As a result, a total of six items were eliminated throughout the process, and the final version of the scale was established with 30 well-aligned items [24].

Following the exploratory factor analysis, confirmatory factor analysis (CFA), a specialized application of structural equation modeling (SEM), was performed to validate the scale’s factor structure and elucidate the interrelations among factors [25]. CFA assessed the alignment of the empirical data with the theoretical framework underpinning the scale’s development, ensuring standardization and theoretical congruence [25–27]. The analysis included evaluating fit indices and modeling the construct relationships via path analysis, employing JASP software for SEM, to rigorously test and confirm the scale’s structural integrity and theoretical validity [25, 27, 28].

### Reliability analysis

In the reliability analysis phase, the internal consistency of the scale was assessed using Cronbach’s alpha and McDonald’s *ω* coefficient, a standard measure for evaluating reliability in scale adaptation studies [29]. McDonald’s *ω* and Cronbach’s alpha value above 0.70 is generally considered acceptable for demonstrating good internal consistency, ensuring that the scale items cohesively measure the same underlying construct [30]. This threshold indicates a reliable scale that can be confidently used for further research and application in the field [31, 32].

**Fig. 1:**
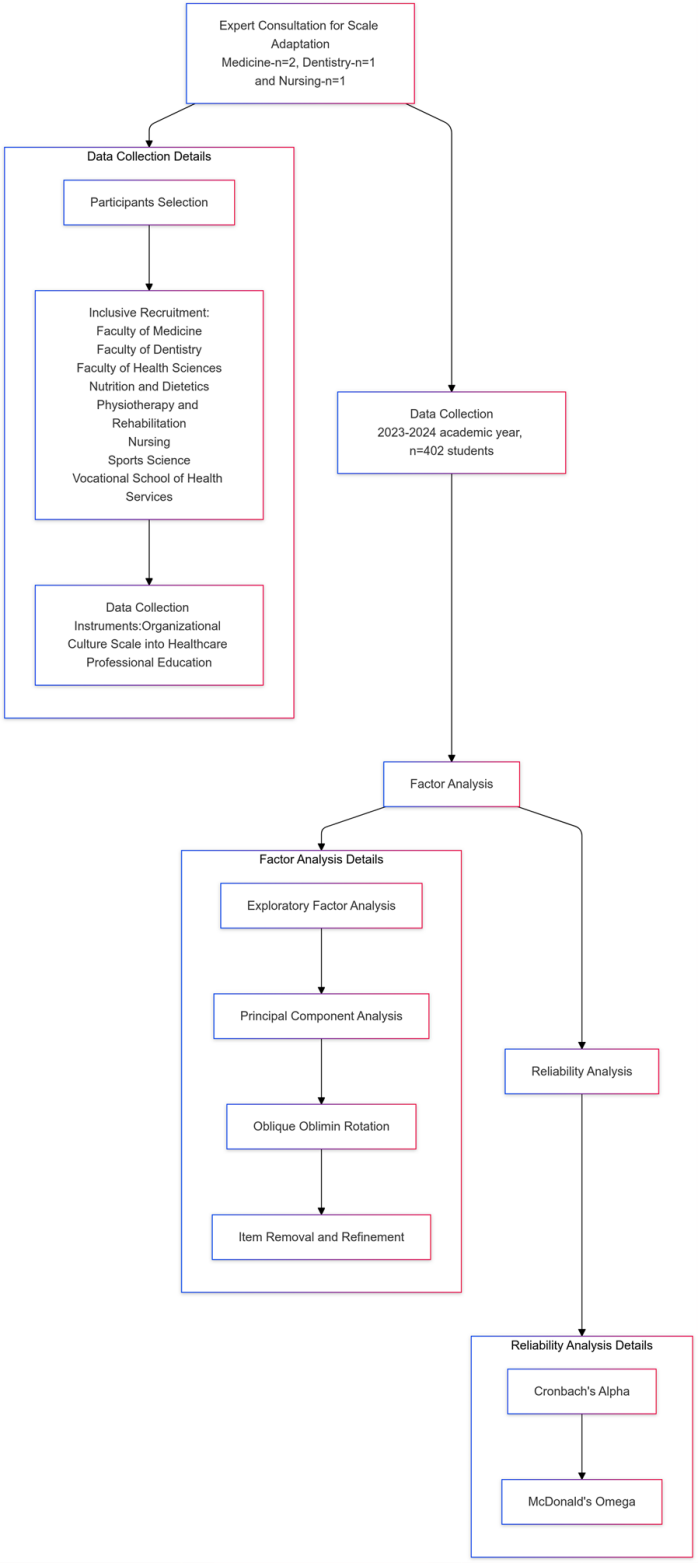
Flowchart of the validity and reliability steps for the OCS

Descriptive statistics (mean values, standard deviations, and percentages) were applied to analyze the demographic attributes of the participants. The data analysis was conducted using SPSS 25.0 and JASP software. To assess the alignment of the adapted scale with the original version, specific criteria were established and applied, as shown in Table 1, to ensure that the adapted scale retained the conceptual and measurement integrity of the OCS (Fig. 1).

**Table 1:** Methods used to ensure conceptual and measurement integrity of the scale

Evaluation aspect	Methodology (reference)
Content validity	Expert consultation [33, 34]
Face validity	Expert consultation [33, 34]
Sample size calculation	Tabachnick ‘tens’ rule [18]
Sample suitability	Kaiser–Meyer–Olkin and Bartlett tests [19]
Construct validity	Exploratory and confirmatory factor analysis [20, 26, 27]
Reliability	McDonald’s omega [32]

## Results

This section presents the main findings regarding the sample characteristics, the content and face validity of the scale, sample size and suitability, and the results of exploratory and confirmatory factor analyses, as well as reliability analysis.

### Demographics

During the 2023–2024 academic year, a total of 402 students from various health-related faculties participated in the study. Among the participants, 66.9% were female. The participants were distributed as follows: 32.1% from the Faculty of Medicine, 12.7% from the Faculty of Dentistry, 49.3% from the Faculty of Health Sciences, and 6% from the Vocational School of Health Services. Within the Faculty of Health Sciences, the student distribution included: Nutrition and Dietetics (15.4%), Physiotherapy and Rehabilitation (18.9%), Nursing (13.7%), and Sports Sciences (5.2%).

### Content and face validity

The consensus among experts was achieved for 34 out of the 36 items on the scale after saturation process statements (numbers 5 and 29) were removed due to redundancy or lack of clarity in their meaning. This refinement process underscored the remaining 34 items’ adequacy in terms of both scope and relevance for assessing organizational culture. Consequently, these 34 items formed the basis for the subsequent phases of the scale adaptation study, ensuring a focused and coherent tool for measuring the organizational culture within educational institutions.

### Sample size and suitability

In adherence to Tabachnick’s ‘‘tens’’ rule, the study collected data from a sample exceeding the minimum required number of participants, ensuring robust statistical analysis. The Kaiser–Meyer–Olkin (KMO) measure and Bartlett’s test of sphericity were deployed to evaluate the sample’s adequacy for factor analysis. The outcomes indicated a KMO coefficient of 0.846, signifying satisfactory sampling adequacy, while Bartlett’s test of sphericity produced a significant Chi-square value (*χ*^*2*^ = 3809.010, df = 435, *p* < 0.001), indicating that the correlation matrix is not an identity matrix and that there are significant relationships among the variables. These results as shown in Table 2 underscore the methodological rigor in assessing the scale’s applicability within the research context.

**Table 2:** KMO and Bartlett’s test results

KMO and Bartlett’s test		
Kaiser–Meyer–Olkin measure of sampling adequacy		0.846
Bartlett’s test of sphericity	Approx. Chi-square	3809.010
	df	435
	Sig	0.000

### Exploratory factor analysis

The exploratory factor analysis revealed the factor characteristics for both unrotated and rotated solutions. In the unrotated solution, Factor 1 had the highest eigenvalue with a sum of squared loadings of 6.99, explaining 23.3% of the total variance. Factor 2 had an eigenvalue of 1.552, accounting for 5.2% of the variance, and Factor 3 had an eigenvalue of 1.4, explaining 4.7% of the variance. The cumulative variance explained by these three factors was 33.1%. Factors 4 and 5 had eigenvalues of 1.13 and 0.925, respectively, with total cumulative variance explained reaching 40%. These results indicate that the first three factors explain a significant portion of the variance in the data, with a gradual decrease in variance explained by the subsequent factors. The rotation has helped in better distribution of variance across the factors, providing a clearer structure and interpretation of the underlying subscales of organizational culture in healthcare professionals education [35].

The exploratory factor analysis (EFA), conducted using the oblimin rotation method, revealed a well-defined structure comprising five distinct factors. Factor 1 (RC1) showed the highest loadings for Item 9 (0.806) and Item 8 (0.714). Factor 2 (RC2) was characterized by strong loadings on Item 35 (0.876) and Item 36 (0.840). Factor 3 (RC3) was primarily represented by Item 21 (0.789) and Item 17 (0.690). Factor 4 (RC4) included notable loadings for Item 12 (0.621) and Item 16 (0.619), while Factor 5 (RC5) showed strong associations with Item 25 (0.653) and Item 13 (0.632). A full summary of item loadings across factors is presented in Tables 3 and 4. The uniqueness values, which reflect the proportion of variance attributed solely to individual items rather than shared among factors, were generally within acceptable ranges. These findings confirm the multidimensional nature of the adapted scale and support its validity for measuring various subscales of organizational culture in healthcare professional education [36–41].

**Table 3:** Total variance explained

Factor characteristics						
	Unrotated solution			Rotated solution		
	SumSq. loadings	Proportion var	Cumulative	SumSq. loadings	Proportion var	Cumulative
Factor 1	6.990	0.233	0.233	2.873	0.096	0.096
Factor 2	1.552	0.052	0.285	2.848	0.095	0.191
Factor 3	1.400	0.047	0.331	2.807	0.094	0.284
Factor 4	1.130	0.038	0.369	1.825	0.061	0.345
Factor 5	0.925	0.031	0.400	1.590	0.053	0.398

**Table 4:** Factor loadings (structure matrix)

	RC1	RC2	RC3	RC4	RC5	Uniqueness
Item9	0.806					0.305
Item8	0.714					0.407
Item10	0.593					0.475
Item28	0.541					0.420
Item11	0.520					0.553
Item4	0.494					0.643
Item27	0.469					0.394
Item30	0.461					0.584
Item35		0.876				0.264
Item36		0.840				0.241
Item34		0.812				0.270
Item32		0.558				0.482
Item23		0.427				0.672
Item21			0.789			0.463
Item17			0.690			0.398
Item18			0.626			0.510
Item19			0.611			0.410
Item24			0.554			0.507
Item20			0.411			0.665
Item12				0.621		0.502
Item16				0.619		0.601
Item7				0.614		0.551
Item15				0.469		0.593
Item1				0.459		0.764
Item6				0.430		0.719
Item3				0.405		0.691
Item25					0.653	0.527
Item13					0.632	0.487
Item26					0.626	0.410
Item22					0.436	0.674

Applied rotation method is oblimin

The exploratory factor analysis, using the oblimin rotation method, identified five distinct factors reflecting thematic content relevant to organizational culture in undergraduate-level institutions. The factors identified through the analysis were aptly named to reflect their thematic content. The factor loadings for each item within their respective factors, along with their minimum and maximum values, are detailed below:

F1: Structural order and formality (including item 9, 8, 10, 28, 11, 4, 27, and 30) factor loadings range from a minimum of 0.461 (Item 30) to a maximum of 0.806 (Item 9).

F2: Belonging and collective responsibility (including item 35, 36, 34, 32, and 23) factor loadings range from a minimum of 0.427 (Item 23) to a maximum of 0.876 (Item 35).

F3: Achievement and performance orientation (including Item 21, 17, 18, 19, 24, and 20) factor loadings range from a minimum of 0.411 (Item 20) to a maximum of 0.789 (Item 21).

F4: Authority and hierarchy (including item 12, 16, 7, 15, 1, 6, and 3) factor loadings range from a minimum of 0.405 (Item 3) to a maximum of 0.621 (Item 12).

F5: Competition orientation (including item 25, 13, 26, and 22) factor loadings range from a minimum of 0.436 (Item 22) to a maximum of 0.653 (Item 25).

These factors collectively explain a significant proportion of the variance in the dataset, indicating a diverse yet coherent set of subscales that underpin organizational culture (Table 4).

### Confirmatory factor analysis

The confirmatory factor analysis is utilizing structural equation modeling indicated a satisfactory fit for the organizational culture scale, as evidenced by various fit indices. A Chi-square to degrees of freedom ratio (*χ*^*2*^/sd) of 3.37 suggests a good model fit. The RMSEA value at 0.091, SRMR at 0.080, along with IFI and CFI both at 0.95, GFI at 0.89, TLI at 0.94, and NFI at 0.93, collectively demonstrate the model’s acceptability. These results from Table 5 affirm the scale’s current structure’s adequacy for addressing the research questions, while also highlighting areas for potential refinement in future iterations.

**Table 5:** Statistical values of fit indexes in structural equation modeling (JASP)

Fit indexes	Excellent fit criteria	Acceptable fit criteria	Fit index obtained
*χ*^*2*^/df	0 ≤ *χ*^*2*^/df ≤ 2	2 <* χ*^*2*^/df ≤ 5	*χ*^*2*^:1329.635/ df:395 = 3.37 *
RMSEA	00 ≤ RMSEA ≤ 0.08	0.08 < RMSEA ≤ 1.00	0.091*
SRMR	00 ≤ SRMR ≤ .05	0.05 < SRMR ≤ .10	0.080*
IFI	0.95 ≤ IFI ≤ 1.00	0.90 ≤ IFI < 0.95	0.95**
CFI	0.95 ≤ CFI ≤ 1.00	0.90 ≤ CFI < 0.95	0.95**
GFI	0.95 ≤ GFI ≤ 1.00	0.90 ≤ GFI < 0.95	0.96**
TLI (NNFI)	95 ≤ TLI ≤ 1.00	0.90 ≤ TLI < 0.95	0.94*
NFI	95 ≤ NFI ≤ 1.00	0.90 ≤ NFI < 0.95	0.93*

*acceptable fit; **excellent fit; source: [25, 27, 28]

CFA and structural equation modeling affirmed the structural coherence of the organizational culture scale, maintaining all 30 items across five defined factors, thereby demonstrating an acceptable fit with the collected data. In the path diagram, illustrated in Supplementary Material, the path coefficients—arrows linking scale items to their corresponding subscales—quantitatively articulate the relationships between the scale’s items and its subscales. This robust validation process reinforces the scale’s construct validity, highlighting its effectiveness in measuring the nuanced aspects of organizational culture. The path diagram illustrates the standardized relationships between the five latent factors (Structural Order and Formality, Belonging and Collective Responsibility, Achievement and Performance Orientation, Authority and Hierarchy, and Competition Orientation) and their associated observed variables (items). Each factor shows strong and positive loadings onto its respective items, confirming the structure identified during exploratory factor analysis. Correlations among the latent factors are also indicated, suggesting moderate interrelationships, which is consistent with the conceptual overlap expected in organizational culture dimensions. Factor loadings mostly exceed 0.40, supporting the convergent validity of the adapted Organizational Culture Scale. The strength of the connections demonstrates that each set of items reliably measures its intended underlying construct (Supplementary Fig. 1).

### Reliability analysis

The reliability of the scale was rigorously evaluated using Cronbach’s *α* 0.874 and McDonald’s omega (*ω*), resulting in a high overall value of 0.878, with a 95% confidence interval ranging from 0.861 to 0.895, indicating strong internal consistency among the scale items. The frequentist reliability statistics are included as a table in Supplementary Material. Factor 1 had an omega of 0.853, Factor 2 had 0.834, and Factor 3 had 0.780, all reflecting good to excellent reliability. However, Factor 4 and Factor 5 showed lower reliability, with omega values of 0.611 and 0.533, respectively, suggesting room for improvement in these factors. Despite this, the overall high omega value underscores the scale’s robustness and reliability in measuring organizational culture within undergraduate-level institutions for healthcare professionals (Supplementary Table 1).

## Discussion

This study highlights the importance of adapting organizational culture frameworks to the specific characteristics of healthcare professional education. İpek’s Organizational Culture Scale, initially designed for general educational contexts, proved highly applicable after validated to reflect the distinct dynamics of healthcare professional education. Factor analysis resulted in a robust five-factor structure with 30 items [provided Turkish (Supplementary Table 2) and English (Supplementary Table 3) translation version as a Supplementary Material], consistent with theoretical expectations and the existing literature. By focusing on the Turkish cultural context and the specific needs of health education, this study offers a novel and comprehensive tool for enhancing the strategic planning and quality improvement efforts in healthcare professional education, a domain previously unexplored with this scale [42].

The factor structure of this adapted scale was supported by strong psychometric properties. The Kaiser–Meyer–Olkin (KMO) value indicated excellent sampling adequacy, confirming that the data were well-suited for factor analysis [43]. Falling within the “well factorizable” range of 0.80–0.90, the KMO result demonstrated that the scale’s items clustered meaningfully into coherent subscales, as recommended in the literature [18]. In addition, the variance ratios and eigenvalues obtained were consistent with established benchmarks for construct validation, reinforcing the structural soundness and theoretical integrity of the adapted OCS [19, 21, 42].

The terminology of the subscales in this adapted scale aligns with prevailing literature [3, 44, 45]. This alignment reinforces the conceptual relevance and practical applicability of the subscales identified: Structural Order and Formality, Belonging and Collective Responsibility, Achievement and Performance Orientation, Authority and Hierarchy, and Competition Orientation. These subscales were derived based on the unique organizational and cultural characteristics of healthcare professional education, which involves not only the acquisition of individual knowledge and skills, but also emphasizes teamwork, interprofessional collaboration, role modeling, and mentor–apprentice dynamics. Given that healthcare professionals are perceived as strong, trustworthy, and exemplary figures in society, the organizational culture shaped during their education plays a critical role in forming their professional identity, ethical values, and collaborative behavior. Moreover, as healthcare institutions inherently require a high level of cooperation and interdisciplinary interaction, the presence of a supportive and participatory culture within educational settings can further strengthen interprofessional relationships. To further elucidate the structure of the adapted scale, each subscale is explained below with reference to its theoretical basis and representative items.

### Structural order and formality

This factor reflects the organization’s structured, planned, and rule-based nature, emphasizing formalities and regulations. Sample items include managers encouraging and rewarding loyalty, meticulously planned and scheduled educational activities, emphasis on completing tasks on time and according to rules, and a collaborative approach to planning educational activities. Literature support indicates that in a bureaucratic culture, rules, procedures, and hierarchy are essential [3, 46–48].

### Belonging and collective responsibility

This factor captures members’ commitment to the organization and their sense of collective responsibility. Sample items include the ease of taking risks for success, a strong sense of responsibility for the faculty’s success, pride in the faculty, protection and defense of the faculty against outsiders, and a strong sense of belonging. Clan culture emphasizes belonging, cooperation, and collective responsibility. In healthcare professional education, fostering a strong sense of belonging among students and staff is essential for developing future professionals who value collaboration and interprofessional cooperation—skills, vital for multidisciplinary healthcare teams [45, 48].

### Achievement and performance orientation

This factor highlights the focus on achievement and performance, with reward systems based on success. Sample items include rewarding job performance, prioritizing results over formalities, supporting and encouraging success, rewarding based on success, and discussing mistakes without focusing on who made them. Achievement culture rewards individual achievement and performance. An achievement-focused culture motivates learners to pursue continuous improvement and adapt to the rapidly evolving demands of healthcare delivery, directly impacting future patient outcomes and professional standards [3, 15, 41, 45, 49].

### Authority and hierarchy

This factor reflects the structured decision-making mechanisms, defined roles, and formalized power relationships that are intrinsic to both healthcare systems and educational institutions that prepare healthcare professionals. In the context of healthcare education, hierarchical structures often mirror clinical environments where authority gradients are prominent—such as between attending physicians, residents, nurses, and students. While such structures are necessary for ensuring accountability, clarity of responsibilities, and patient safety in clinical settings, they may also influence educational dynamics. For example, strong hierarchical cultures can sometimes hinder open communication, discourage student participation, or suppress critical questioning—all of which are essential for active learning and professional development. Conversely, when authority is exercised transparently and combined with supportive mentorship, it can reinforce professional identity, foster respect for institutional norms, and prepare students for the realities of hierarchical healthcare systems. Therefore, understanding how authority and hierarchy are perceived and enacted within educational institutions is crucial for balancing discipline with empowerment, and tradition with innovation, in the education of future healthcare professionals [3, 46, 48, 50].

### Competition and success orientation

This factor captures the degree to which individuals and institutions emphasize achievement, performance, and comparison-based motivation within organizational culture. In healthcare professional education, this orientation manifests in various ways—through academic rankings, competitive examination systems, performance-based evaluations, and prestige-driven program structures. While a moderate level of competition can drive motivation, excellence, and accountability among students, excessive emphasis on outperforming peers may hinder collaborative learning, foster stress, and undermine team-based competencies that are essential in healthcare environments. Therefore, striking a balance between fostering personal achievement and promoting shared responsibility is essential in shaping a healthy educational culture. Recognizing and assessing this cultural dimension can guide institutions in aligning successful metrics with professional values, ensuring that students not only strive for excellence, but also internalize the cooperative spirit necessary for effective healthcare delivery [45, 47, 50].

During the adaptation process, certain items emphasizing excessive competitiveness or rigid bureaucratic control were excluded to better align the scale with contemporary academic realities. The refinement of the scale reflects a conscious adjustment to the nuanced environment of healthcare professional education, where collaboration, academic freedom, and flexible achievement pathways are increasingly prioritized. This careful modification ensures that the adapted scale captures dimensions of organizational culture that are both meaningful and relevant to healthcare professional education. This validated instrument offers a multidimensional framework that reflects critical aspects of educational culture, including authority and hierarchy, structural formality, belonging, performance orientation, and competition. Its application extends beyond mere cultural assessment—providing valuable insights for various institutional stakeholders. For students, the scale can help reveal how organizational culture shapes their learning experiences, sense of professional identity, and engagement within interprofessional and hierarchical environments. For faculty members, it offers a lens through which to evaluate their role as institutional role models, contributors to cultural climate, and facilitators of collaborative learning. For administrators and institutional leaders, the instrument serves as a tool to assess cultural alignment with strategic goals, inform curriculum reform, and guide organizational development.

The structural validity of the adapted OCS was further confirmed through confirmatory factor analysis (CFA), employing structural equation modeling techniques. The CFA demonstrated that the model achieved satisfactory fit indices, with values for RMSEA, SRMR, IFI, CFI, GFI, TLI, and NFI all falling within acceptable thresholds [26, 27]. Although the confirmatory factor analysis (CFA) results generally indicated an acceptable model fit, it is important to acknowledge that the RMSEA value slightly exceeded the commonly accepted threshold of 0.08, and the GFI value fell marginally below the optimal criterion of 0.90 [51]. This slight deviation may be attributed to the limitations of sample size distribution across faculties; a more proportionally stratified sampling approach—ensuring at least 5–10 participants per item from each faculty—might have yielded more optimal fit indices**.** Nonetheless, the overall model fit remains robust, and the results are considered sufficiently strong to support the scale’s structural validity. Future studies with larger and more diverse samples can further validate and refine the model. These deviations suggest that while the model is acceptable, there is potential for improvement. Future model refinements could include re-examining modification indices to identify and address localized areas of misfit, such as correlating residuals between theoretically related items or exploring item parceling strategies to reduce model complexity [30]. Additionally, further cross-validation of the model with independent samples from different healthcare professional education could help verify the scale’s structure and enhance its generalizability. Structural modifications, such as allowing for covariance between closely related subscales or reevaluating items with lower factor loadings, may also yield improved fit indices while maintaining theoretical integrity complexity [30].

The adapted OCS demonstrated a strong degree of internal consistency, reflecting its reliability as a measurement tool for organizational culture in healthcare professional education. Compared to the original version of İpek’s scale, which had acceptable reliability the adapted version exhibited an enhanced level of cohesion across both the overall scale and its subscales. This outcome confirms that the adapted items consistently measure the underlying cultural constructs they intend to capture, offering researchers and practitioners a dependable instrument for organizational assessment. The improved reliability strengthens the scale’s ability to support empirical studies and institutional evaluations aimed at enhancing undergraduate-level education.

Overall, the findings align with established literature emphasizing that scales reaching or exceeding the reliability threshold of 0.70 are considered methodologically sound [32]. The successful adaptation and validation process reinforce the robustness of the OCS in capturing the multifaceted nature of organizational culture in healthcare professional education.

OCS offers practical applications for a range of stakeholders within healthcare professional education institutions. Policymakers can use the scale to systematically assess the existing cultural environment and identify areas where targeted reforms are necessary to foster a more collaborative, achievement-oriented, or student-centered institutional culture. Faculty developers can apply OCS results to design professional development programs that align faculty behaviors and attitudes with the institution’s desired cultural attributes, such as promoting innovation, teamwork, or accountability. Administrators can utilize the scale findings to inform strategic decisions in curriculum planning, ensuring that teaching methods, assessment strategies, and interprofessional education initiatives are culturally congruent and supportive of the institution’s broader mission. Moreover, longitudinal use of the scale can help administrators monitor cultural shifts over time following major institutional changes such as curriculum reforms, mergers, or accreditation processes.

While the study provides valuable insights into organizational culture within healthcare professional education, several limitations must be acknowledged. Cross-sectional design limits the ability to assess causal relationships or track changes in organizational culture over time; future research should incorporate longitudinal designs to monitor cultural evolution across academic years. Moreover, voluntary participation may have introduced self-selection bias, as participants may differ systematically from non-respondents, potentially limiting the broader generalizability of the findings. Another important limitation is the use of the same dataset for both exploratory and confirmatory factor analyses, which increases the risk of overfitting; future studies are recommended to implement split-sample validation or to use independent samples for EFA and CFA separately. Additionally, cross-cultural adaptations and validation in different educational contexts, such as pharmacy, public health, and veterinary education, are suggested to enhance the external validity and applicability of the adapted OCS across diverse settings.

## Conclusion

In conclusion, this study has yielded a psychometrically sound and contextually relevant adaptation of the Organizational Culture Scale for use in healthcare professional education. The scale’s application is recommended for tracking cultural changes over time and adjusting educational designs accordingly. In addition to monitoring cultural dynamics over time, the scale can support the evaluation and improvement of educational programs, ensuring that they foster inclusive, accountable, and professionally nurturing environments. Future studies may utilize the scale for cross-institutional comparisons and longitudinal validation to further expand its utility and impact within the field of healthcare education. Future uses of the scale should include confirmatory factor analyses to ensure ongoing validity .

## Supplementary Information

Supplementary material 1.

## Data Availability

The datasets supporting the conclusions of this article are available in the Mendeley data repository through 10.17632/3k4bdm6w7m.2 the preprint version of this article is available on Research Square at: https://www.researchsquare.com/article/rs-3970831/v1.

## Abbreviations

CFA: Confirmatory factor analysis
CFI: Comparative fit index
DOI: Digital object identifier
EFA: Exploratory factor analysis
GFI: Goodness-of-fit index
IFI: Incremental fit index
JASP: Jeffreys’s amazing statistics program
KMO: Kaiser–Meyer–Olkin measure
NFI: Normed fit index
OCS: Organizational culture scale
RMSEA: Root mean square error of approximation
SEM: Structural equation modeling
SPSS: Statistical Package for the Social Sciences
SRMR: Standardized root mean square residual
TLI: Tucker–Lewis index
UK: United Kingdom
