# Supplementary material for: Adapting organizational culture scale into healthcare professional education: a scale validity and reliability analysis
Source: Hum Resour Health. 2025 Jul 15;23:33. doi: 10.1186/s12960-025-01006-2 (PMC12265199; doi:10.1186/s12960-025-01006-2)
Supplement: Supplementary file 1 — Supplementary material 1. [file 12960_2025_1006_MOESM1_ESM.docx]

Supplementary Materials

**Supplementary Table 1: Frequentist Scale Reliability Statistics**

| **Frequentist Scale Reliability Statistics** | **F1** | **F2** | **F3** | **F4** | **F5** | **Scale** |
| --- | --- | --- | --- | --- | --- | --- |
| **Estimate** | **McDonald's ω** | **McDonald's ω** | **McDonald's ω** | **McDonald's ω** | **McDonald's ω** | **McDonald's ω** |
| Point estimate | 0.853 | 0.834 | 0.780 | 0.611 | 0.533 | 0.878 |
| 95% CI lower bound | 0.831 | 0.809 | 0.747 | 0.553 | 0.46 | 0.861 |
| 95% CI upper bound | 0.875 | 0.86 | 0.813 | 0.669 | 0.606 | 0.895 |

Supplementary Figure 1: Path Diagram of the modelling (output of JASP 0.16.4.0)


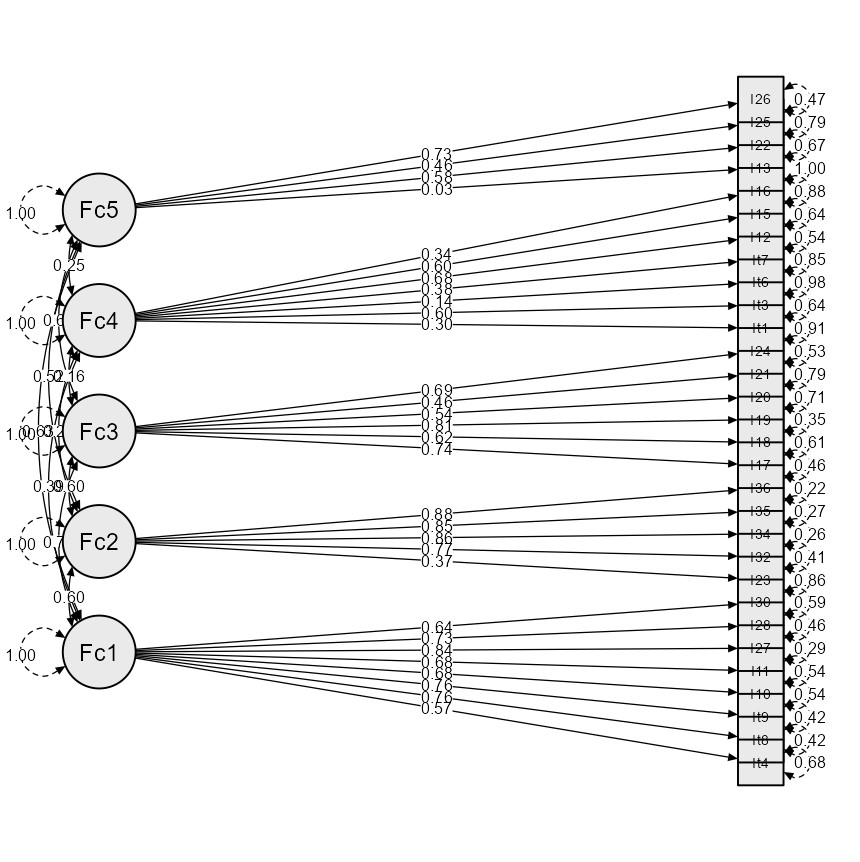


**Supplementary Table 2: Final Adapted Organizational Culture Scale (OCS) — Turkish Version**

| **Factor** | **Item No** | **Item (Turkish)** |
| --- | --- | --- |
| **Yapısal Düzen ve Resmiyet** | 9 | İşlerin zamanında ve kurallara göre yapılmasına önem verilir. |
|  | 8 | Eğitim-öğretim faaliyetleri ayrıntılı planlanıp programlanır. |
|  | 10 | Herkesin her anı planlanmıştır. |
|  | 28 | Eğitim-öğretim faaliyetleri işbirliği ile planlanır. |
|  | 11 | Herkesin ve her şeyin yeri bellidir. |
|  | 4 | Yöneticiler sadakati teşvik eder ve ödüllendirir. |
|  | 27 | Alınan kararlar, doğru ve yeterli bilgiye dayandırılır. |
|  | 30 | Yönetim, her seviyeden gelen fikir ve önerilere açıktır. |
| **Aidiyet ve Kolektif Sorumluluk** | 35 | Herkes okulu dışa karşı korur ve savunur. |
|  | 36 | Herkes kendini okulun bir parçası olarak görür. |
|  | 34 | Herkes okuluyla gurur duyar. |
|  | 32 | Herkes okulun başarısı için sorumluluk duyar. |
|  | 23 | Herkes, başarılı olmak için rahatlıkla risk üstlenebilir. |
| **Başarı ve Performans Yönelimi** | 21 | Yanlışı kimin yaptığı değil, sonuçları tartışılır. |
|  | 17 | Herkes işini iyi yapmanın karşılığını alır. |
|  | 18 | Formalitelerden çok, sonuca önem verilir. |
|  | 19 | Başarı desteklenir ve teşvik edilir. |
|  | 24 | Kişisel bilgi ve yetenekler ön planda tutulur. |
|  | 20 | Ödüllendirmede başarı esas alınır. |
| **Otorite ve Hiyerarşi** | 12 | Resmi ilişkiler ön plandadır. |
|  | 16 | İlişkilerde hiyerarşik yapı esas alınır. |
|  | 7 | Anlaşmazlıklar yönetimin isteği doğrultusunda çözülür. |
|  | 15 | Yöneticiler, sık sık kurallara uyulmasını hatırlatır. |
|  | 1 | Hiç kimse yönetimle ters düşmek istemez. |
|  | 6 | Ast-üst arasındaki ilişkiler çok resmidir. |
|  | 3 | Değişim ve yenilikler yönetimce başlatılır. |
| **Rekabet Yönelimi** | 25 | Güçlü bir rekabet söz konusudur. |
|  | 13 | Yönetim, asıl işlerden çok ayrıntılarla uğraşır. |
|  | 26 | Okulun başarı düzeyi velilerce de izlenir. |
|  | 22 | Herkes başarı düzeyinin yüksek olmasını ister. |

**Supplementary Table 3: Final Adapted Organizational Culture Scale (OCS) — English Translation Version**

| **Factor** | **Item No** | **Item (English Translation)** |
| --- | --- | --- |
| **Structural Order and Formality** | 9 | Importance is placed on completing tasks on time and according to the rules. |
|  | 8 | Educational activities are planned and scheduled in detail. |
|  | 10 | Everyone’s every moment is scheduled. |
|  | 28 | Educational activities are planned collaboratively. |
|  | 11 | Everyone and everything has a designated place. |
|  | 4 | Managers encourage and reward loyalty. |
|  | 27 | Decisions are based on accurate and sufficient information. |
|  | 30 | Management is open to ideas and suggestions from all levels. |
| **Belonging and Collective Responsibility** | 35 | Everyone protects and defends the institution externally. |
|  | 36 | Everyone sees themselves as part of the institution. |
|  | 34 | Everyone takes pride in their institution. |
|  | 32 | Everyone feels responsible for the institution’s success. |
|  | 23 | Everyone can take risks easily for success. |
| **Achievement and Performance Orientation** | 21 | The focus is on the outcomes, not on who made a mistake. |
|  | 17 | Everyone receives recognition for doing their job well. |
|  | 18 | Results are prioritized over formalities. |
|  | 19 | Success is supported and encouraged. |
|  | 24 | Personal knowledge and skills are emphasized. |
|  | 20 | Success is the basis for rewards. |
| **Authority and Hierarchy** | 12 | Formal relationships are emphasized. |
|  | 16 | Hierarchical structure is the basis of relationships. |
|  | 7 | Conflicts are resolved according to management’s preferences. |
|  | 15 | Managers frequently remind employees to comply with the rules. |
|  | 1 | No one wants to come into conflict with management. |
|  | 6 | Relationships between subordinates and superiors are very formal. |
|  | 3 | Changes and innovations are initiated by management. |
| **Competition Orientation** | 25 | There is strong competition. |
|  | 13 | Management focuses more on details than main tasks. |
|  | 26 | The institution’s success is monitored by external stakeholders (e.g., parents). |
|  | 22 | Everyone desires a high level of success. |
